# Metabolic changes in the midgut of Eri silkworm after Oral administration of 1-deoxynojirimycin: A ^1^H-NMR-based metabonomic study

**DOI:** 10.1371/journal.pone.0173213

**Published:** 2017-03-01

**Authors:** Ming-Jie Deng, Xiao-Dong Lin, Chao-Wei Wen, Min-Jian Dong, Qiu-Ting Lin, Shang-Zhi Zhang, Jia-Ping Xu

**Affiliations:** 1 Analytical and Testing Center of Wenzhou Medical University, Wenzhou, China; 2 School of Life Sciences, Anhui Agricultural University, Hefei, China; Institute of Plant Physiology and Ecology Shanghai Institutes for Biological Sciences, CHINA

## Abstract

1-deoxynojirimycin (DNJ) is a natural D-glucose analogue and has a strong physiological activity in inhibiting α-glucosidase *in vivo*. The antidiabetic effects of DNJ in mice or other mammals were extensively explored, but the physiological and toxic roles of DNJ in insects was seldom reported. In this study, the biological effects of DNJ were examined in midgut extracts of fourth-instar larvae of Eri silkworm (*Samia cynthia ricini*, Saturniidae). Based on nuclear magnetic resonance (NMR) metabonomics technology, we analyzed the alterations of glycometabolism, lipids, and energy metabolism pathways in the midgut of *S*. *cynthia ricini* caused by DNJ. Pattern recognition analysis (partial least square-discriminant analysis, PLS-DA) showed that four groups of latex, 0.25% DNJ, 0.5% DNJ and the mixture of 0.5% DNJ and latex (1:1) were distinctly different from the control group. Moreover, several metabolic pathways of DNJ-mediated modulation in the midgut were identified. Compared with the control group, alanine, succinate, glutamate, and fumarate concentrations decreased in three groups of 0.5% DNJ, latex, and the mixture, choline levels increased in two DNJ groups, and trehalose levels increased in all experimental groups. Therefore, these results suggest that DNJ modulated lipid metabolism by limiting the hydrolysis pathways of phospholipids metabolism. Additionally, DNJ has a potent negative effect on energy metabolism by inhibiting the hydrolysis of trehalose, glycolysis and the tricarboxylic acid (TCA) cycle. Overall, DNJ, as a single-ingredient, is an efficient substance for modulating lipid metabolism and inhibiting energy metabolism.

## Introduction

Mulberry leaves have been widely cultivated for rearing the silkworms, *B*. *mori*, from ancient times. However, mulberry trees are not the natural host plants of Eri silkworm, *S*.*cynthia ricini*. Mulberry leaves exude latex, containing the rich defense protein MLX56 and alkaloids, such as 1-deoxynojirimycin (DNJ) [1, 2]. These active substances are lethal to Eri silkworms and *Mamestra brassicae* Linnaeus, a generalist, herbivorous insect [3].

DNJ, a 5-amino-1,5-deoxy-D-glucopyranose, is the most abundant iminosugar in mulberry and has a chemical structure similar to that of glucose [4]. As the most competitive inhibitor of intestinal α-glucosidases, this D-glucose analogue has been receiving a great deal of attention [5]. Due to the low cytotoxicity, DNJ has been explored in the treatment of many metabolic diseases, such as hyperglycemia, obesity and diabetes [6]. Its biological applications appear to be extended to treat patients with Type-2 diabetes mellitus (T2DM), Gaucher’s diseases and hepatitis [7–9]. These studies suggest that the potential bioactivity of DNJ needs to be evaluated in detail. Though sericulture products and their extracts have been reported for antidiabetic effect [6], there has been no irrefutable evidence of the hypoglycemic efficacy of DNJ, isolated from sericulture products as a single compound in an animal model. Therefore, it is necessary to investigate the physiological perturbations associated with the bioactivity of DNJ, which, in turn, may facilitate the identification of DNJ utility associated with health effects.

Like mammals, Eri silkworms have a special mechanism to regulate hemolymph sugar and material energy [10]. Larva is the only feeding stage in the growth and development of silkworm, so the larva stage is the most important stage in the whole life cycle of silkworms and has substantial influences on the metabolism and life activities of the metamorphosis [11–13]. Hemolymph is a fluid in the blood cavity of insects and analogous to the blood in vertebrates. The main physiological effects of hemolymph include nutrients transportation, temperature regulation, and wound healing [14]. Our previous study revealed the impaired hydrolytic pathway of trehalose and tricarboxylic acid (TCA) cycle in the hemolymph of fourth-instar larvae after oral administration of DNJ [15]. The hemolymph worked as a metabolic bridge between the various organs of silkworms, so we reasonably suspected that DNJ could also cause metabolic disturbance in tissues. The midgut is, not only the main location of nutrient digestion and absorption, but also the initial opposing front of external signal molecules in silkworms [10, 16]. Additionally, the silkworm midgut has the physiological function of modifying dietary lipids, and it is the fundamental organ where a silkworm reserves the formation of tissue in an organized manner [17]. The metabolic regulation of glucose levels and lipid storage in the intestines of mammals is parallel to the regulation of carbohydrates and lipid availability in the midgut of silkworm larvae [10, 18]; metabonomic research using the midgut of silkworm larvae is a manageable option. In the study, we explored the metabolite changes in multiple metabolic pathways based on midgut ^1^H NMR spectra of silkworms, after oral administration of DNJ or mulberry latex, in comparison with their age-matched controls. The results were consistent with the consequences of hemolymph metabonomics of silkworms in our previous work [15].

Nuclear magnetic resonance (NMR) can achieve non-destructive, non-selective, and non-biased analysis of samples. Also it can be carried out under near-physiological conditions, and the experimental methods are also flexible [19]. A metabonomic analysis method, which is based on NMR, is one of the main technologies used in current metabolomics and it is suitable for the study of the complex components in metabolites, the pathology research, and mechanism improvement research after drug action [20–22]. Based on its usefulness in estimating subtle metabolic perturbations caused by systemic responses, metabonomics can be applied to the physiological evaluation of DNJ and the identification of its potential medicinal value [23, 24].

In the present study, we evaluated the roles of DNJ in lipid metabolism and its effects on phospholipid-metabolism-mediated metabolic changes in the midgut of Eri silkworm, *S*. *cynthia ricini*. To this end, we investigated whether DNJ influenced energy metabolism and glycometabolism in the midgut of Eri silkworm, which ultimately exerted biological effects against these processes by inhibiting trehalose hydrolysis, glycolysis, and tricarboxylic acid (TCA) cycle in the midgut. Furthermore, we examined the effects of DNJ on protein and branched-chain amino acid (BCAA) metabolism pathway associated with metabolic disorders in the midgut of Eri silkworm.

## Material and methods

### Insects

*S*. *cynthia ricini* larvae were purchased from the Sericultural Research Institute of the Chinese Academy of Agricultural Sciences (Jiangsu University of Science and Technology, Zhenjiang) and then maintained at the Animal Experimental Center of Wenzhou Medical University (Wenzhou). In this study, newly molted fourth-instar *S*. *cynthia ricini* were adopted in order to evaluate the effects of mulberry latex and DNJ. Castor oil leaves were consumed ad libitum by hatched larvae in the entire experiment. The experimental conditions in the animal laboratory were maintained as follows: the light-dark cycle of 12 h: 12 h, room temperature of 22 ± 3°C, and relative humidity of 50~70%. Administration procedures for the silkworms were performed according to the handbook of laboratory animal care and use of Wenzhou Medical University.

### Mulberry latex

**Fig 1:**
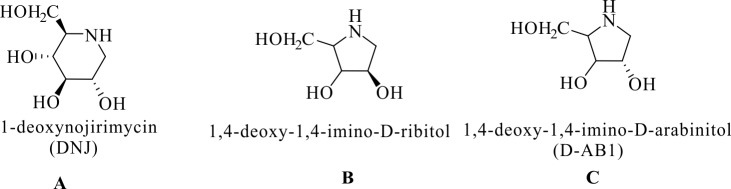
Molecular structures of 1-deoxynojirimycin (A), 1,4-dideoxy-1,4-imino-D-ribitol (B), and D-AB1 (C).

*Morus alba* was cultivated from the botanical gardens of Wenzhou Medical University, Wenzhou, China. In this work, mulberry latex was collected from *Morus alba* by directly cutting petioles. The administration procedures of mulberry were approved by the Institutional Plant Committee and Use Committee of Wenzhou Medical University (Document No.: wydw2012-0083). Only 0.32 ± 0% of DNJ was detected in the latex of this population, and there were no traces of 1,4-dideoxy-1,4-imino-D-ribitol and D-AB1 (Fig 1) [1]. The latex from *Morus alba* was collected in test tubes, preserved at 4°C, and then used within 12 h.

### Experimental design and sample collection

Firstly, 200 newly-molted fourth-instar larvae were randomly divided into four groups, and then 5-μL of latex, 0.25% DNJ (J&K Chemicals, Beijing, China), 0.5% DNJ, and the mixture of 0.5% DNJ and latex (1:1) were respectively fed to each larve of the four groups. The feeding liquid was dropped to mouthparts by using pipette (Eppendoff, Hamburg, Germany), and larvae would drink the liquid within 30 s. In addition, 5 μL of ultrapure water (Millipore, Massachusetts, USA) was fed to 50 larvae as the control group. After continuous administration for 2 d, individuals from each group were dissected and the midgut was collected on day 3. Midguts from 5 individuals were mixed together as one sample. The collected midguts were immediately snap-frozen in liquid nitrogen and stored at –80°C until ^1^H NMR analysis.

### Preparation of midgut samples and acquisition of ^1^H NMR spectra

Midgut tissue samples were prepared according to our previous method [25]. After weighing the frozen tissues, water-soluble small-molecule metabolites were obtained using a methanol–chloroform–water extraction method [26]. Firstly, corresponding midgut tissues were placed in EP tubes and ground using an electric homogenizer with ultrapure water (0.85 mL/g) and ice-cold methanol (4 mL/g) at 4°C, and then the mixture was vortexed for 15 s. Secondly, after the addition of ultrapure water (2 mL/g) and chloroform (2 mL/g), the mixture was vortexed again. After 15-min ice bath, the tissue homogenate was centrifuged for 15 min (1000 g, 4°C). Finally, water-soluble metabolites in the supernatant were extracted and lyophilized for 24 h.

The freeze-dried midgut powder was then dissolved in 0.6 mL of 99.5% D_2_O, and then vibrated for 15 s for complete dissolution. The dissolved tissue was centrifuged for 10 min (1000 g, 4°C). Then, 500 μL of supernatant was extracted for NMR spectroscopy (Bruker, Munich, Germany). All ^1^H NMR experiments were implemented on a Bruker AVANCE III 600 MHz NMR spectrometer with a sampling number of 64 K, scan time of 256 and a spectral width of 12,000 Hz. Prior to Fourier transformation, the spectra were zero-filled to 64 K, and an exponential line-broadening function of 0.3 Hz was applied to the free induction decay. All of the spectra were calibrated for phase correction and baseline adjustment using Topspin (v2.1 pl4, Bruker Biospin, Munich, Germany), and the methyl peak of alanine (CH3, 1.48 ppm) was used to perform the calibration [27].

### Data refinement and multivariate pattern recognition analysis

In order to explore the metabolites contained in the NMR spectra, using the Topspin 2.1 software package, each spectrum (δ9.5~0.5 ppm) was segmented into the same width (0.01 ppm) as the interval for automatic segmentation. The δ5.0~4.6 region was removed to zero with the aim of eliminating the distortion caused by the residual peaks from water resonance. Before multivariate analysis, Microsoft Excel was used to import the data of the remaining spectral segments, and then the peaks of the spectra were normalized. In the analysis, the relevant peak areas represented the concentrations of metabolites. After normalization, the integral value of 0.01 ppm was exported to SIMCA-P 12.0 (Umetrics, Umea, Sweden) for pattern recognition analysis. The integral peak area value of 0.0015 ppm was imported into SPSS 13.0 (SPSS Inc., Chicago, USA) for statistical analyses.

In order to differentiate the metabolic profiles obtained from the midgut samples of the five groups, partial least square-discriminant analysis (PLS-DA) was adopted in order to process the data obtained from the midgut samples. The X and Y axes of PLS-DA respectively represented the two principal components, PC2 and PC1. Each point on the score plots denotes a sample. Based on PLS-DA results, the quality of the data model and the relative intensity of the metabolites were evaluated. The loading plots could complement related score plots. The loading plots exhibited metabolites related to differences among these groups. The parameters of R^2^ and Q^2^ were computed to test the goodness of fit and model validity, where R^2^ represented the sum of the square of the entire X and Q^2^ was the fraction of cross-validation-explained variation with the increase in reliability [28, 29].

### Statistical analyses

The SPSS 13.0 software package was used for statistical analysis and the data were expressed as mean ± standard deviations (SD). Each experimental group was compared with the control group. In the statistical analyses, the acquired data from two groups were analyzed using the independent-samples t-test. If the calculated *P*-value was lower than 0.05, the difference was believed to be statistically significant.

## Results

### Security of DNJ

To examine the defensive effect of DNJ derived from mulberry latex, the roles of DNJ and latex in Eri silkworm, *S*. *cynthia ricini*, were analyzed. The latex from *Morus alba* contains 0.32 ± 0% of DNJ and excludesD-AB1 and 1,4-dideoxy-1,4-imino-D-ribitol [1]. The DNJ concentrations in the five groups were gradually decreased in the following sequence: 0.5% DNJ, the mixture of latex and 0.5% DNJ, latex, 0.25% DNJ, and H_2_O. After oral administration of latex containing 0.32% DNJ, the survival rate was greatly decreased to 60% and other Eri silkworms displayed the significantly retarded growth and frequent vomiting. However, no death was observed in other groups (n = 50 in each group), regardless of the concentration of DNJ or other compounds. Our results showed that the toxicity of mulberry latex is completely dependent on its own unidentified high-molecular-weight (UHMW) factor(s) and the defense protein MLX56 [1, 30, 31]; the concentrations of UHMWs and MLX56 accounted for the toxicity of latex; DNJ had no obvious toxicity to *S*. *cynthia ricini* over a short time (Table 1).

**Table 1:** Concentrations of DNJ in five groups of four-instar *S*. *cynthia ricini* and their mortality rate, respectively.

Groups	Quantity	Concentrations of DNJ (%)	Mortality rate (%) on Day 3
H_2_O	50	0	0
0.25% DNJ	50	0.25	0
0.5% DNJ	50	0.5	0
latex	50	0.32	40
Mixture	50	0.41	0

### ^1^H NMR spectra and pattern recognition analysis of midgut extracts

**Fig 2:**
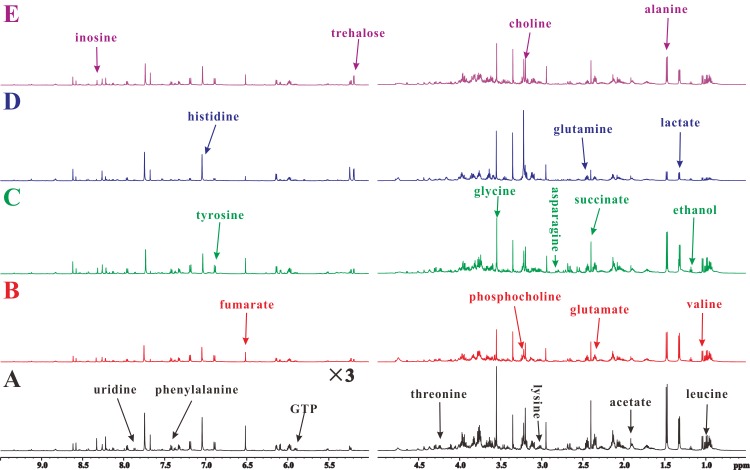
Representative ^1^H NMR spectra of midgut extracts obtained from a water-fed Eri silkworm (A), a 0.25% DNJ-fed Eri silkworm (B), a 0.5% DNJ-fed Eri silkworm (C), a latex-fed Eri silkworm (D), and a mixture-fed Eri silkworm (E).

Representative ^1^H-NMR spectra of midgut extracts, acquired from Eri silkworms, in the control and 0.25% DNJ, 0.5% DNJ, latex and the mixture groups are respectively shown in Fig 2A–2E. According to our previous work [32, 33], the 600 MHz library of the Chenomx NMR suite 7.0 (Chenomx Inc., Edmonton, Canada) was used to assign the spectral resonances of the metabolites. The inspection of some samples was carried out by using 2D ^1^H–^1^H COSY spectra with solvent suppression to verify the assignments from 1D ^1^H NMR spectra. Endogenous metabolites, such as leucine (δ0.94), valine (δ1.03), lactate (δ1.33), alanine (δ1.47), acetate (δ1.91), glutamate (δ2.34), succinate (δ2.39), glutamine (δ2.45), asparagine (δ2.83), lysine (δ3.02), choline (δ3.2), phosphocholine (δ3.22), glycine (δ3.55), threonine (δ4.23), trehalose (δ5.18), GTP (δ5.93), fumarate (δ6.51), tyrosine (δ6.88), phenylalanine (δ7.42), histidine (δ7.04), uridine (δ7.86), and inosine (δ8.31), were simultaneously measured through the ^1^H-NMR spectra of the midgut extracts (Table 2).

**Table 2:** Metabolite normalized intensity of midgut extracts obtained from newly molted forth-instar larvae controls and age-matched experimental Eri silkworms, *S*. *cynthia ricini*.

δ ^1^H (ppm)	Metabolite	H_2_O	0.25% DNJ	0.5% DNJ	Latex	Mixture of 0.5% DNJ and Latex
**0.94**	leucine	4.68 ± 0.51	4.93 ± 0.22	4.41 ± 0.26	1.92 ± 0.37**	3.66 ± 0.43**
**1.03**	valine	3.91 ± 0.36	4.47 ± 0.14**	4.48 ± 0.40**	1.34 ± 0.38**	2.88 ± 0.24**
**1.33**	lactate	3.97 ± 1.25	3.55 ± 0.96	2.94 ± 1.54	1.64 ± 1.44*	1.77 ± 1.10**
**1.47**	alanine	13.18 ± 2.01	11.48 ± 0.93	10.09 ± 0.64**	4.44 ± 0.48**	9.17 ± 0.74**
**1.91**	acetate	2.17 ± 0.12	2.43 ± 0.17**	2.13 ± 0.19	0.97 ± 0.48*	1.93 ± 0.21*
**2.34**	glutamate	13.25 ± 0.60	11.55 ± 0.49**	12.04 ± 0.43**	8.17 ± 1.29**	10.97 ± 0.76**
**2.39**	succinate	3.04 ± 0.49	2.70 ± 0.25	2.52 ± 0.25**	1.77 ± 0.39**	2.49 ± 0.29**
**2.45**	glutamine	12.10 ± 1.11	10.43 ± 0.58**	11.34 ± 0.77	11.48 ± 0.61	7.81 ± 0.54**
**2.83**	asparagine	3.56 ± 0.63	3.30 ± 0.51	3.55 ± 0.47	2.27 ± 0.22**	2.63 ± 0.26**
**3.02**	lysine	3.69 ± 0.21	3.94 ± 0.18**	3.93 ± 0.22**	4.07 ± 1.43	3.80 ± 0.27
**3.2**	choline	14.44 ± 1.90	11.54 ± 0.98**	11.74 ± 1.03**	15.06 ± 3.80	15.54 ± 1.57
**3.22**	phosphocholine	1.45 ± 0.20	1.51 ± 0.14	1.33 ± 0.26	9.66 ± 1.34**	1.87 ± 0.31**
**3.55**	glycine	18.70 ± 1.69	15.31 ± 0.47**	16.77 ± 1.49*	26.08 ± 1.32**	19.13 ± 2.81
**4.23**	threonine	8.96 ± 0.41	9.22 ± 0.24	9.61 ± 0.37**	6.54 ± 1.31**	8.13 ± 0.67*
**5.18**	trehalose	0.34 ± 0.18	1.05 ± 0.26**	1.40 ± 0.53**	3.71 ± 0.59**	1.90 ± 0.71**
**5.93**	GTP	0.28 ± 0.05	0.30 ± 0.02	0.29 ± 0.02	0.50 ± 0.09**	0.37 ± 0.08*
**6.51**	fumarate	2.25 ± 0.29	1.69 ± 0.15**	1.91 ± 0.23**	0.75 ± 0.24**	1.70 ± 0.11**
**6.88**	tyrosine	3.23 ± 0.29	3.65 ± 0.17**	3.54 ± 0.29*	1.87 ± 0.23**	3.35 ± 0.25
**7.42**	phenylalanine	2.38 ± 0.27	2.69 ± 0.13*	2.25 ± 0.19	1.38 ± 0.12**	1.82 ± 0.18**
**7.04**	histidine	5.88 ± 0.40	5.76 ± 0.37	5.62 ± 0.49	9.90 ± 1.28*	5.63 ± 0.53
**7.86**	uridine	0.61 ± 0.07	0.52 ± 0.05*	0.46 ± 0.05**	0.70 ± 0.07	0.47 ± 0.04**
**8.31**	inosine	1.32 ± 0.30	0.80 ± 0.21**	0.81 ± 0.21**	0.49 ± 0.17**	1.18 ± 0.29

Values are expressed as mean ± SD.

*p < 0.05 and

**p < 0.01 respectively indicate significant differences and extremely significant differences compared with the control group.

**Fig 3:**
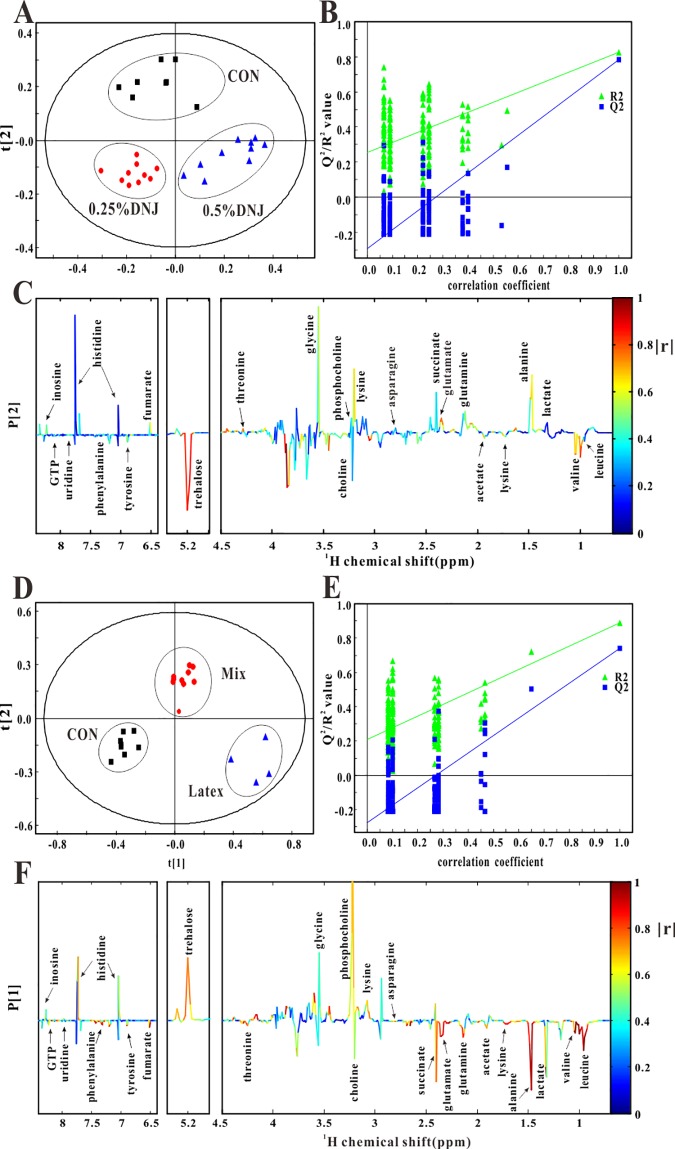
Results of partial least square-discriminant analysis (PLS-DA) of ^1^H NMR spectra of midgut samples. PLS-DA score plots (A) based on ^1^H NMR spectra of midgut extracts from Eri silkworms of the 0.25% DNJ group, 0.5% DNJ group, and control group (R^2^X = 0.424, R^2^Y = 0.84, Q^2^ = 0.768, black square: control group, red circle: 0.25% DNJ group, blue triangle: 0.5% DNJ group), validation plot (B) and coefficient-coded loading plots (C). PLS-DA score plots (D) based on ^1^H NMR spectra of midgut extracts from Eri silkworms of the latex group, mixture group, and control group (R^2^X = 0.518, R^2^Y = 0.922, Q^2^ = 0.828, black square: control group, red circle: mixture group, blue triangle: latex group), validation plot (E) and coefficient-coded loading plots (F).

Partial least square-discriminant analysis (PLS-DA) was applied to determine more details about the segmented NMR spectra of the midgut, in which the changes in various metabolic systems and potential metabolic pathways associated with the effects of DNJ were observed (Fig 3). As shown in Fig 3A, the 0.25% DNJ and 0.5% DNJ groups could be clearly discriminated in the direction of PC1 (R^2^X = 0.424, R^2^Y = 0.84, Q^2^ = 0.768). Additionally, these two groups were separated from their age-matched controls along the PC2 direction, indicating that the cluster of 0.25% DNJ and 0.5% DNJ groups had unequal metabolite profiles compared to the control group. Fig 3B showed the validation plot of permutation analysis, which specified the reliability and robustness of PLS-DA model. In the corresponding loading plot between DNJ groups and the control group (Fig 3C), the color-coded correlation coefficients (|r|) of metabolites suggested the variables causing the separation of the different groups. The positive regions of the loading plot were equal to the decreased levels of metabolites in the midgut of Eri silkworms, whereas the negative regions amounted to elevated concentrations of metabolites in the midgut of Eri silkworms. Thus, corresponding loading plot (Fig 3C) indicated that the separation pertained to various metabolites, including trehalose, fumarate, glycine, alanine lysine, valine, glutamate, and acetate.

For further analyses, the separation of midgut extracts of latex, mixture, and the control groups were demonstrated by the PLS-DA score plot, and these groups were disjointed along the PC1 direction or PC2 direction (Fig 3D, R^2^X = 0.518, R^2^Y = 0.922, Q^2^ = 0.828). The clear separation of latex and the mixture groups from the age-matched control group proved that latex and mixture actuated the metabolic perturbations in Eri silkworms. The validation plot of permutation test was shown in Fig 3E. Corresponding loading plot (Fig 3F) showed thathistidine, trehalose, glycine, phosphocholine, choline, succinate, glutamate, glutamine, alanine, lactate, valine, and leucine were the most variables responsible for the separation.

### Metabolite concentrations in midgut extracts

**Fig 4:**
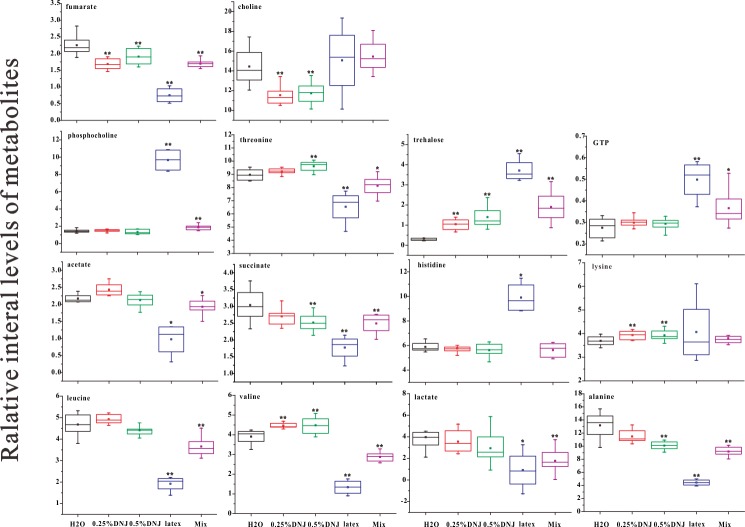
Box plots of relative integral levels of main metabolites in midgut samples of different groups of Eri silkworms. *p < 0.05 and **p < 0.01 respectively indicate significant differences and extremely significant differences compared with the control group.

Table 2 reveals the corresponding metabolite levels in the midgut samples of Eri silkworms of different groups. Quantitative statistical analyses (Table 2) indicated the trend of the metabolic variances in Eri silkworms, which were consistent withthose shown in the PLS-DA loading plots (Fig 3). Box plots (Fig 4, S2 Fig) were prepared for typical metabolite concentrations of midgut samples in order to verify the discerned physiological effects of DNJ treatment on Eri silkworms. These results (Fig 4, S2 Fig) showed that trehalose was extremely elevated in the midgut of Eri silkworms after oral administration of DNJ, latex, or mixture, suggesting the inhibition of trehalase and the utilization of trehalose, the main sugar in insects. Two appended glycolysis-relevant products, lactate and alanine, were decreasd in the midgut after feeding latex and mixture. As the primary tricarboxylic acid (TCA) cycle intermediates, the midgut extracts of succinate, fumarate was reduced in four of the treatment groups to different degrees, and achieved a minimum in the latex group. As the substrates of gluconeogenesis, glutamine and glutamate were reduced in the midgut of the mixture, latex, 0.25% DNJ and 0.5% DNJ groups. The excretion of acetate, valine, threonine, tyrosine, and phenylalanine was enhanced in the 0.25% DNJ and 0.5% DNJ groups, but the excretion of the above substances in the mixture and latex group was decreased. As a branched-chain amino acid (BCAA), leucine was considerably decreased in the latex and mixture groups. In addition, the results demonstrated that 0.25% DNJ and 0.5% DNJ decreased the lipid metabolites of choline, and the latex and mixture increased phosphocholine. Compared to the control group, the levels of histidine, uridine, and inosine were notably decreased in the 0.25% DNJ and 0.5% DNJ groups. GTP was enhanced in the mixture and latex groups. The level of lysine was enhanced in the 0.25% DNJ and 0.5% DNJ groups, and asparagine was decreased in the latex and mixture groups. The level of glycine was elevated in the latex group and decreased in the 0.25% DNJ and 0.5% DNJ groups.

## Discussion

**Fig 5:**
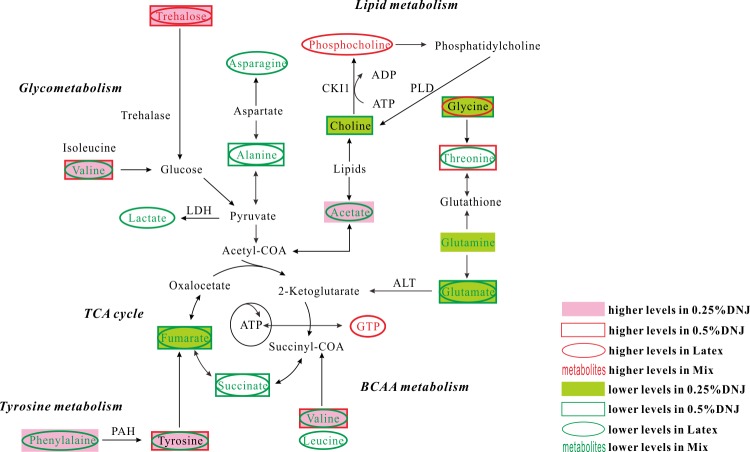
Schematic diagram of the metabolic pathways. The metabolite changes detected with ^1^H NMR midgut analysis, and the pathway referenced to the KEGG database showed the interrelationships of the identified metabolic pathways in the experimental Eri silkworms. Metabolites with increased and decreased levels in the midgut, compared with those in the control group, are respectively indicated in red and green.

NMR is a useful method for investigating tissue energy metabolism, glycometabolism, and lipid metabolism, and it has been utilized to analyze the modulations in tissue metabolisms of diverse diseases, such as encephalopathies, nephropathy, and intestinal cancer [34–36]. In the current study, the modulations in the levels of midgut metabolites in Eri silkworms fed with H_2_O, mixture, latex, 0.25% DNJ, and 0.5% DNJ were explored with *ex vivo* ^1^H NMR spectroscopy. Conspicuous metabolic disorders of biochemicals, including some foundational products of glycometabolism, energy, and lipid metabolism were detected. DNJ-induced changes in the concentrations of metabolites showed transformational characteristics of metabolic pathways in the mixture, latex, 0.25% DNJ, and 0.5% DNJ groups. Based on previous results, the metabolites and corresponding pathways modulated by DNJ might serve as underlying metabolic biomarkers for therapeutic targets and illuminate the molecular mechanisms of the agents acting on hypoglycemia [5, 6, 9, 37]. Fig 5 indicates the changed metabolic pathways in the midgut of Eri silkworms after oral administration of the mixture, latex, 0.25% DNJ, and 0.5% DNJ based on the KEGG database (http://www.genome.jp/kegg/pathway.html).

### Lipid metabolism

As the main location of nutrient digestion and absorption, the silkworm midgut has the physiological function to modify dietary lipids, and it is the fundamental organ of reserving the substances in an organized manner [10, 17]. The present study contains a biochemical investigation of midgut extracts in order to estimate the changes in lipid metabolism after oral administration of latex DNJ and the mixture. NMR analyses showed the reduced levels of choline in the 0.25% DNJ and 0.5% DNJ groups as well as elevated concentrations of phosphocholine in the latex and mixture groups. Choline is the precursor hydrolysis product of phospholipids. In a hydrolysis process catalyzed by phospholipase D, phosphatidylcholine is primarily transformed into phosphatidic acid and choline [38]. Simultaneously, as an important synthetic intermediate of phosphatidylcholine in tissues, phosphocholine is produced and catalyzed by choline kinase in a reaction of inverting ATP and choline into phosphocholine and ADP [39]. Decreased levels of choline in the midgut extracts may be related to the impaired hydrolysis pathways of phospholipid metabolism in Eri silkworms fed with 0.25% DNJ and 0.5% DNJ. Increased phosphocholine concentrations in the latex and mixture groups suggested enhanced activity of choline kinase in Eri silkworms. These results demonstrated that, with DNJ, there was a significant difference in terms of the latex and mixture groups in the phosphocholine pathways in Eri silkworms. It was speculated that single-ingredient DNJ had a suppressive function in the hydrolysis pathways of phospholipids, while latex and mixture provided additional beneficial effects in phosphocholine synthesis and that UHMWs might play a major role. In addition, acetate, as end product of fatty acid oxidation, is mainly utilized by organisms in the formation of acetyl-coenzyme A (acetyl-CoA), and the decreased acetate level in the latex and mixture groups indicated a suppressive acetyl-CoA pool caused by both exogenous (bacterial fermentation) and endogenous factors (mammalian) [40, 41]. To sum up, DNJ modulated lipid metabolism by limiting the hydrolysis pathways of phospholipid metabolism, whereas latex and the mixture had an enhanced regulation effect on phosphocholine synthesis, and primarily relied on the UHMWs within them. DNJ groups showed a significant difference compared with the mixture and latex groups in the regulated pathways of lipid metabolism.

### Glycometabolism and energy metabolism

Glycometabolism is crucial for the physiological homeostasis of living organisms. In insects, trehalose is the major sugar and metabolic source of energy in hemolymph, and the hydrolysis of trehalose in two glycosidically linked glucose units is catalyzed by trehalase [42]. Due to its importance in insect physiology, trehalose is a good target for insect studies on glycometabolism and energy metabolism. In this study, the increased levels of trehalose, which was related to glycometabolism, were observed in the midgut extracts of 0.25% DNJ-, 0.5% DNJ-, latex-, and mixture-fed Eri silkworms. This increase in midgut trehalose of DNJ groups, was likely ascribed to a low elemental metabolic rate of carbohydrates at the stage. This finding suggested that DNJ played a central role in the adjustment of trehalose metabolism, and might reduce trehalase activity in the midgut due to trehalase hydrolyzing only trehalose [43]. The elevated trehalose level might be derived from the impaired hydrolysis of trehalose to glucose in midgut, thereby reducing trehalose transport into the hemolymph. In insects, trehalose exists in many tissues, where it supplies energy and plays a critical role in energy metabolism and coping with stress [44]. An increased concentration of trehalose suggested that trehalose utilization was obstructed after absorption in the midgut, tindicating that the metabolic pathway of trehalose could be a limiting factor in glycometabolism in all the experiment groups compared to the control group.

The tricarboxylic acid (TCA) cycle is a succession of chemical reactions in all aerobic organisms to generate chemical energy in the form of adenosine triphosphate (ATP) through the oxidation of acetate, which originates from proteins, carbohydrates, and fats, into carbon dioxide [45, 46]. The TCA cycle is the most effective way to get energy from carbohydrates or other substances that are oxidized in organisms. Its central importance in many biochemical pathways suggests that it is the hinge of carbohydrate, fat, and protein metabolism. In the study, the reduced levels of succinate and fumarate, associated with the TCA cycle, were detected in midgut extracts of Eri silkworms in 0.5% DNJ, latex, and the mixture groups. This suggested that the pathways of the TCA cycle and aerobic metabolism were impaired after oral administration of latex, DNJ and the mixture [47]. It is known that these metabolites are intermediates of energy synthesis, as a result, the decreased metabolites might induce lower production of ATP [32]. Therefore, we hypothesized that curtailing the bioenergy metabolism in Eri silkworms by feeding with DNJ, latex and the mixture might be a significant event involving the disturbed mitochondrial function and aerobic metabolism pathways.

The lessening concentrations of alanine and lactate in the midgut are indicative of depressed glycolysis in Eri silkworms in the latex and mixture groups. Reduced concentrations of glutamate and valine were observed in Eri silkworms in the groups of DNJ, latex, and the mixture, which illustrated the impairment of gluconeogenesis and the reduced pathway of glycogenic amino acid metabolism [48]. In addition, as an important source of energy, guanosine triphosphate (GTP) was increased significantly in the latex and mixture groups. This finding also indicated the suppressed production of ATP in Eri silkworms fed with latex and mixture, as GTP was able to convert to ATP with nucleoside-diphosphate kinase (NDK) and generate one molecule of ATP [49]. Based on the analysis results mentioned above, DNJ, latex, and the mixture might reduce the level of glycometabolism by inhibiting the TCA cycle and glycolysis, and impairing gluconeogenesis in the midgut of Eri silkworms. In this way, the production of ATP and the energy metabolism level declined.

The alterations in pathways, namely, alanine, aspartate, and glutamate metabolism, as indicated in our pathway analysis results (Fig 5), could also be a consequence of an impaired mitochondrial metabolism, because metabolites involved in the energy metabolism are also important intermediates of the pathways mentioned above.

### Other metabolic changes

Tyrosine and lysine are important intermediates of a variety of biological substances involved in tyrosine metabolism and protein synthesis. Enhanced levels of tyrosine and lysine in the midgut observed in the DNJ groups suggested an increased pathway of tyrosine metabolism and protein synthesis in Eri silkworms fed with DNJ. Levels of branched-chain amino acids (BCAAs), such as valine and leucine, considerably decreased in the midgut extracts of Eri silkworms in the experimental groups compared to age-matched control group. This finding revealed that the pathway of BCAA metabolism was disturbed in silkworms after oral administration of latex, DNJ and the mixure. In the mixture and latex groups, we detected the attenuated level of asparagine, which was an important precursor in the ornithine cycle and in amino acid synthesis [50, 51]. Moreover, other metabolites, such as glycine, threonine, phenylalanine, histidine, uridine, and inosine, related to fat metabolism, transamination, neurotransmitters, energy, and nucleoside metabolism, were disturbed in all experimental groups. Nevertheless, the metabolic tendency of these metabolites in the DNJ groups was opposite to that in the mixure group and latex group; The peculiar mechanisms will be explored in the next stage.

## Conclusion

In this work, NMR-based midgut metabonomic analyses were performed in order to evaluate the effects of DNJ on Eri silkworms and elucidate underlying molecular mechanisms. The two-day DNJ and latex treatments were significantly induced metabolic perturbations in the midgut of Eri silkworms by intervening in some dominating metabolic pathways, including lipid, carbohydrate, energy and BCAA metabolism. Our results preliminarily indicated that DNJ was an efficient medicine for regulating glycometabolism, energy, and lipid metabolism.

## Supporting information

S1 Fig Results of principal component analysis (PCA) of ^1^H NMR spectra of midgut extracts. PCA score plots (A) based on ^1^H NMR spectra of midgut extracts from Eri silkworms of 0.25% DNJ group, 0.5% DNJ group and control group (black square: control group, red circle: 0.25% DNJ group, blue triangle: 0.5% DNJ group). PCA score plots (B) based on ^1^H NMR spectra of midgut extracts from Eri silkworms of latex group, mixure group and control group (black square: control group, red circle: mixture group, blue triangle: latex group).
(TIF)

S2 Fig Box plots of relative integral levels of residual metabolites absented from Fig 3. (TIF)
