# Supplementary figures and images for: Metabolic changes in the midgut of Eri silkworm after Oral administration of 1-deoxynojirimycin: A 1H-NMR-based metabonomic study
Source: PLoS One. 2017 Mar 1;12(3):e0173213. doi: 10.1371/journal.pone.0173213 (PMC5332107; doi:10.1371/journal.pone.0173213)

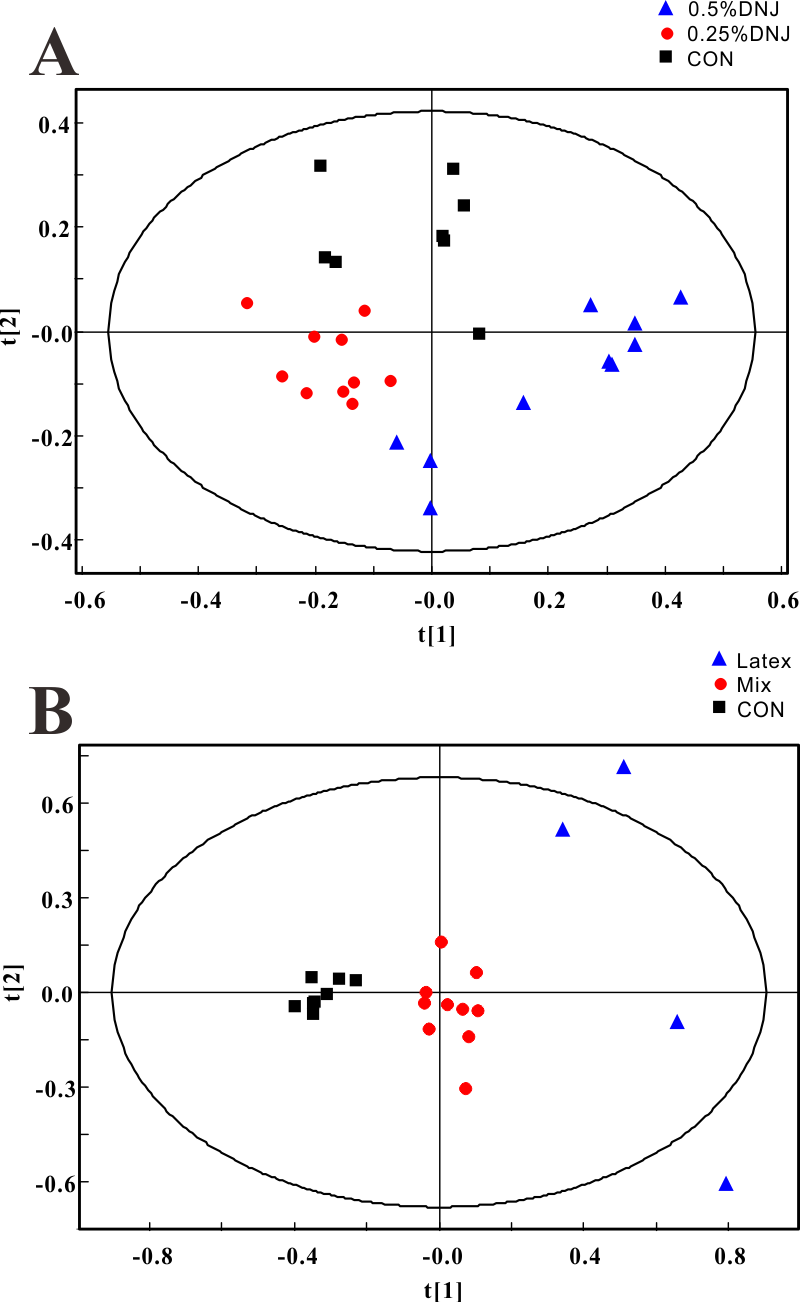

Supplement: S1 Fig — PCA score plots (A) based on 1H NMR spectra of midgut extracts from Eri silkworms of 0.25% DNJ group, 0.5% DNJ group and control group (black square: control group, red circle: 0.25% DNJ group, blue triangle: 0.5% DNJ group). PCA score plots (B) based on 1H NMR spectra of midgut extracts from Eri silkworms of latex group, mixure group and control group (black square: control group, red circle: mixture group, blue triangle: latex group). (TIF) [file pone.0173213.s001.tif]

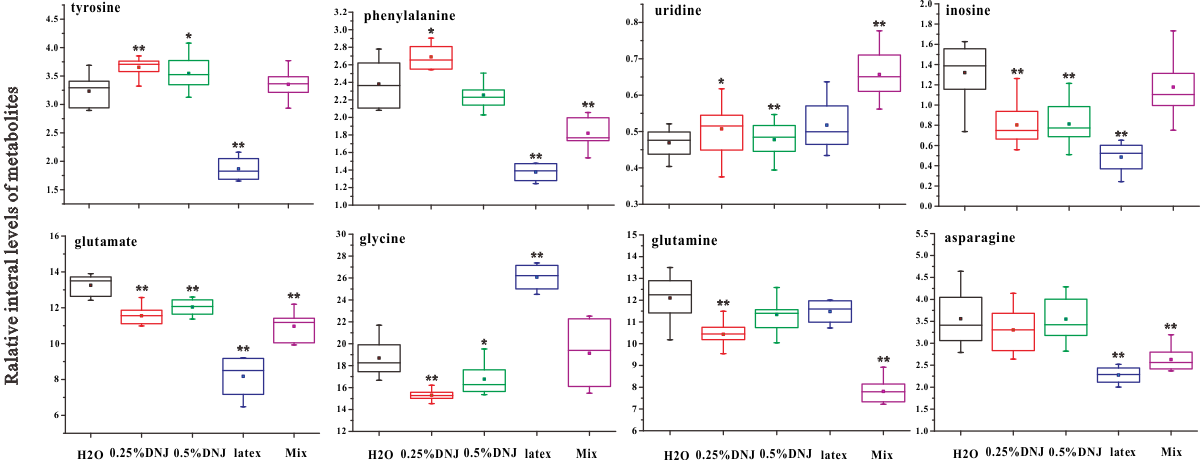

Supplement: S2 Fig — (TIF) [file pone.0173213.s002.tif]
